# Investigation of SARS-CoV-2 Infection among Companion Animals in Households with Confirmed Human COVID-19 Cases

**DOI:** 10.3390/pathogens13060466

**Published:** 2024-06-01

**Authors:** Heather Venkat, Hayley D. Yaglom, Gavriella Hecht, Andrew Goedderz, Jennifer L. Ely, Michael Sprenkle, Taylor Martins, Daniel Jasso-Selles, Darrin Lemmer, Jordan Gesimondo, Irene Ruberto, Kenneth Komatsu, David M. Engelthaler

**Affiliations:** 1 Arizona Department of Health Services, Phoenix, AZ 85007, USA; gavriellahecht@gmail.com (G.H.); irene.ruberto@azdhs.gov (I.R.); ken.komatsu@azdhs.gov (K.K.); 2 Career Epidemiology Field Officer Program, Center for Preparedness and Response, Centers for Disease Control and Prevention, Atlanta, GA 30329, USA; 3 Translational Genomics Research Institute, Pathogen and Microbiome Division, Flagstaff, AZ 86005, USAdlemmer@tgen.org (D.L.); dengelthaler@tgen.org (D.M.E.); 4 Independent Veterinarian, Glendale, AZ 85308, USA

**Keywords:** SARS-CoV-2, companion animals, pets, genomic sequencing, One Health, surveillance

## Abstract

We aimed to characterize SARS-CoV-2 infection in companion animals living in households with COVID-19-positive people and understand the dynamics surrounding how these animals become infected. Public health investigators contacted households with at least one confirmed, symptomatic person with COVID-19 for study recruitment. Blood, nasal, and rectal swab specimens were collected from pet dogs and cats and a questionnaire was completed. Specimens were tested for SARS-CoV-2 by RT-PCR, and for neutralizing antibodies; genomic sequencing was performed on viral-positive samples. A total of 36.4% of 110 pets enrolled had evidence of infection with SARS-CoV-2. Pets were more likely to test positive if the pet was immunocompromised, and if more than one person in the home was positive for COVID-19. Among 12 multi-pet households where at least one pet was positive, 10 had at least one other pet test positive. Whole-genome sequencing revealed the genomes of viral lineages circulating in the community during the time of sample collection. Our findings suggest a high likelihood of viral transmission in households with multiple pets and when pets had very close interactions with symptomatic humans. Further surveillance studies are needed to characterize how new variants impact animals and to understand opportunities for infection and spillover in susceptible species.

## 1. Introduction

The impact of SARS-CoV-2 on animals has been increasingly recognized since the start of the pandemic. Natural infection in animals, such as pets and captive wildlife, is believed to occur through close contact with infected humans [1,2,3,4,5]. Previous literature has shown that animals do not play a major role in spreading the virus to people (zoonotic transmission), although low-level animal-to-human transmission of SARS-CoV-2 has been documented at mink farms and in encounters with white-tailed deer, cats, and lions [6,7,8,9]. Zoonotic transmission and changes in virus variants can be better investigated using early and sustained animal surveillance [10,11].

For example, Chile reported the first detection of the Omicron BA.4.1 variant in 2022 in a dog and a cat due to conducting a COVID-19 household surveillance study; this variant was circulating among people in the area at that time [12]. Further linking people and pets, another surveillance study in the United States during the beginning of the COVID-19 pandemic investigated domestic animals (dogs and cats) that had frequent daily contact (>1 h) with an index patient who tested positive for SARS-CoV-2 infection; more dogs and cats with continued contact with the index patient tested seropositive for SARS-CoV-2 than dogs and cats who had decreased contact with the index patient after the person’s diagnosis [13]. The importance of investigating SARS-CoV-2 in pets using households is further strengthened by the findings of a study in Switzerland during 2020; the prevalence of SARS-CoV-2 infection in dogs and cats presenting to veterinary clinics was low even in pandemic hotspot regions, while more recent studies on animals in COVID-19 households found a higher prevalence of infection [14].

Both dogs and cats can become infected with SARS-CoV-2, but clinical illness varies and can range from asymptomatic to mild respiratory illness, to more severe disease and even death in rare cases [1,4,15,16,17]. Although cats were found to have higher susceptibility to SARS-CoV-2 compared to dogs in a 2023 systematic review and meta-analysis of previous studies performed in America, no significant association between cats and dogs was found in Europe; the conclusion was that additional epidemiological and experimental studies on the seroprevalence of SARS-CoV-2 in dogs and cats is necessary [18]. It is clear that continued One Health surveillance efforts are needed to understand the human–animal–environment interplay with SARS-CoV-2 epidemiology and evolution.

In order to conduct One Health surveillance for SARS-CoV-2 in Arizona, the Arizona Department of Health Services (ADHS) and Translational Genomics Research Institute (TGen) collaborated to create the Arizona COVID-19 and Pets Program (AZCPP) in 2021. The AZCPP study was conducted to (1) characterize SARS-CoV-2 infection in companion animals living in households with COVID-19-positive people and (2) understand the dynamics surrounding how these animals become infected.

## 2. Materials and Methods

The study was approved by the Human Subjects Review Board of the Arizona Department of Health Services (protocol #20-0017 on 11 December 2020) and was approved by TGen’s Institutional Animal Care & Use Committee (protocol #20163 on 17 November 2020).

### 2.1. Recruitment and Sample Collection

People with confirmed COVID-19 were identified using Arizona’s Infectious Disease Surveillance System (MEDSIS); a line-list from MEDISIS was downloaded for all people with confirmed COVID-19 who tested RT-PCR-positive and were symptomatic (had at least 2 symptoms, including fever, cough, loss of taste/smell) within the past 14 days. Households in which at least one person had confirmed, symptomatic COVID-19 were contacted by phone by public health staff for study recruitment. Once households verbally consented to enrollment, trained veterinary and public health staff visited to obtain written consent; collect blood, nasal, and rectal swab specimens from pets; and complete a questionnaire about pet demographics and interactions with the owner(s). Sampling occurred during March–December 2021, when initially B.1.1.7 (Alpha), and then B.1.617.2 (Delta), AY.44 (Delta), AY.103 (Delta), and, lastly, BA.1 (Omicron) were the predominantly circulating variants in Arizona as the months passed.

### 2.2. RT-PCR, Serology, and Genomic Sequencing

Nasal and rectal swab specimens were tested for SARS-CoV-2 by real time polymerase chain reaction (RT-PCR). SARS-CoV-2 genomic material was extracted from nasal swab samples using Zymo DNA/RNA extraction kits (Zymo Research Corporation, Irvine, CA, USA). The extracted material was tested using an RT-PCR assay, developed and validated in-house, which targets the N and S protein of the virus. Oligonucleotide primers and dual-labeled hydrolysis probes (TaqMan^®^, ThermoFisher Scientific, Waltham, MA, USA) were the reagents used. The samples were screened on the Bio-Rad CFX96 instrument (Bio-Rad Laboratories, Hercules, CA, USA) after cDNA synthesis and denaturation; cycle threshold (Ct) values were generated to determine qualitative results. A sample was deemed positive if the Ct values for both targets were 38 or below. Serum was centrifuged at 2500 rpm for 10 min, aliquoted, and tested for viral neutralizing antibody presence using the GenScript cPass™ Neutralizing Antibody Assay (GenScript Diagnostics Technology Co., Nanjing City, Jiangsu Province, China) following the manufacturer’s instructions [19].

Genomic sequencing was performed on positive RT-PCR samples with Ct values ≤ 38 using previously described methods [20,21,22]. Viral genomic data were processed using TGen’s Amplicon Sequencing Analysis Pipeline [22], and SARS-CoV-2 lineages were characterized using PANGOLIN (https://cov-lineages.org/resources/pangolin.html, accessed on 10 May 2023) [23]. The sequencing data were further processed through Nextstrain, the genomes were aligned to the Wuhan-Hu-1 reference genome (EPI_ISL_402125), and a maximum likelihood phylogenetic tree was generated using IQ-Tree (http://www.iqtree.org/, version 2.3.4, accessed on 10 May 2023) [24]. A subset of 35 SARS-CoV-2 genomes from COVID-19-positive people collected within the same geographic region and time period matching the lineages identified in pets was downloaded from GISAID for genomic context. Viral genomes with breadth of coverage < 95% were excluded from phylogeny. The final phylogeny was visualized with metadata using MicrobeTrace (https://microbetrace.cdc.gov/MicrobeTrace/, accessed on 10 May 2023) [25].

### 2.3. Data Analysis

Epi Info™ (version 7) and SAS (version 9.3) were used to conduct univariate and multivariate analyses of pet and human questionnaire data, including laboratory testing results. We compared proportions to estimate the relative risk of PCR-positive or seropositive samples using Fisher’s exact test. We categorized household COVID-19 transmission risk levels for COVID-19 transmission from people to pets in the household based on the vaccination status of the residents: Low (all individuals are fully vaccinated), Medium (at least one individual is not fully vaccinated), and High risk (all individuals are unvaccinated). We used the U.S. Centers for Disease Control and Prevention’s 2021 definition of a fully vaccinated individual, specifying that it had been at least 2 weeks after receiving the final dose in the primary series of any commercial COVID-19 vaccine. In addition, dog and cat data were combined because the rates for many variables were similar. The dog and cat data were also combined in order to make easier recommendations for people regarding their pets in general, rather than potentially how to interact with cats and dogs differently. Combining data for both dogs and cats also aligns with other published studies regarding SARS-CoV-2 and pets.

## 3. Results

### 3.1. Descriptive Results

During March–December 2021, a total of 110 companion animals (39 cats and 71 dogs) were sampled across 45 households (Table 1). Forty (36.4%) companion animals tested positive for SARS-CoV-2 (PCR positive, seropositive, or both). Among the 40 SARS-CoV-2-positive animals, 27 were dogs and 13 were cats. The majority of seropositive pets were dogs (71% of 31).

Moreover, 77.8% of the SARS-CoV-2-positive dogs were asymptomatic while 46.2% of the SARS-CoV-2-positive cats were asymptomatic. Among the 33 symptomatic SARS-CoV-2-positive pets, the reported symptoms in the dogs with clinical signs were limited to sneezing, lethargy, and diarrhea, while more extensive upper respiratory symptoms were reported in the cats with clinical signs. All symptomatic pets eventually recovered from their illness.

Among 12 multi-pet households where at least one pet was positive, 10 had at least one other pet test positive. Veterinary care was obtained for 6/110 (5.5%) pets, but none had been tested for SARS-CoV-2 during their veterinary visit. In addition, 73% of pets (80) sampled resided in Maricopa County, followed by 26% (29) from Coconino County and 1% (1) from Navajo County, Arizona. Among 19 pets with PCR-positive samples, 14 tested positive through nasal swabs, 4 tested positive through rectal swabs, and for 1, both nasal and rectal swabs tested positive. No humans were reported to have become ill after having contact with any of the pets.

### 3.2. Whole-Genome Sequencing

**Figure 1:**
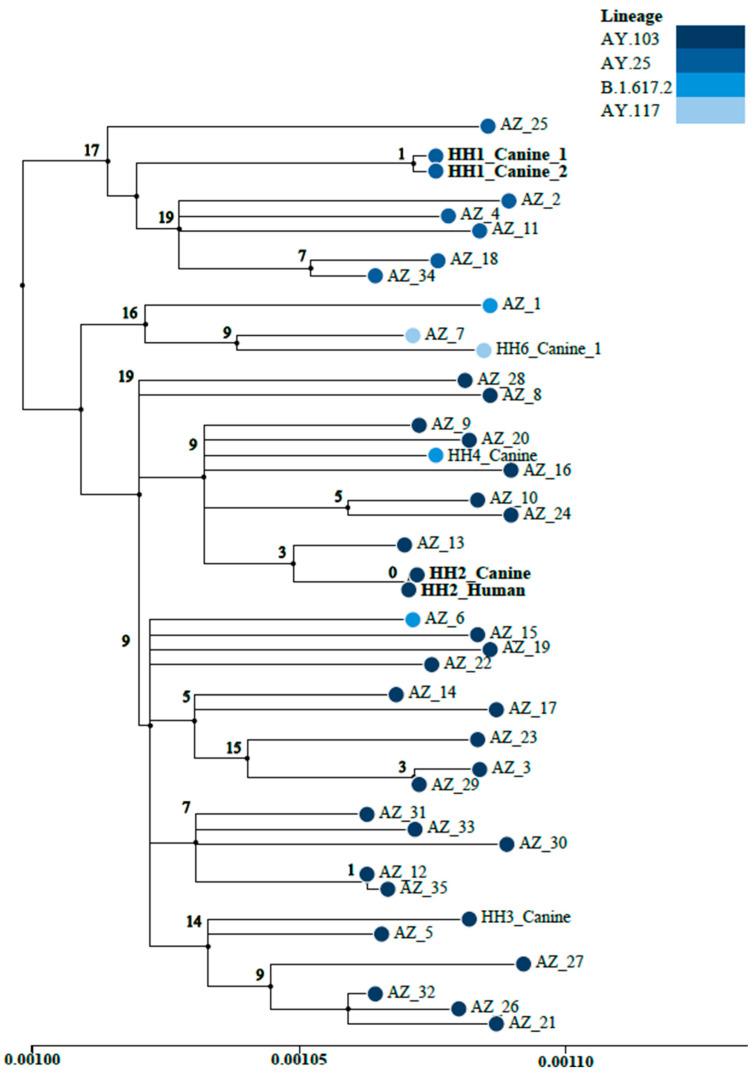
Maximum likelihood phylogenetic tree (QBC: 97.6%) of Delta variant SARS-CoV-2 genomes sequenced from COVID-19-positive pets enrolled in AZCPP, pet owners, and 35 SARS-CoV-2 genomes derived from human COVID-19-positive samples collected within the same geographic region and timeframe for context. Phylogeny node coloration represents Delta variant SARS-CoV-2 lineages (AY.103, AY.25, B.1.617.2, and AY.117) from pets, pet owners, and contextual Arizona genomes. The genomically related samples from HH1 [AY.25] and HH2 [AY.103] are bolded. Associated metadata, GISAID IDs, and Delta PANGOLIN lineages are available in Supplementary Table S1.

Whole-genome sequencing performed on PCR-positive samples yielded SARS-CoV-2 genomes of the B.1 (1), B.1.575 (2), B.1.1.7/Alpha (1), B.1.617.2/Delta (2), AY.25.1/Delta (2), AY.103/Delta (2), and AY.117/Delta (2) lineages. Infection in one cat corresponded to the Alpha variant, while infection in seven dogs and one cat corresponded to the Delta variant. These variants of concern were also circulating in the Arizona community, as illustrated by the maximum likelihood phylogenetic tree of SARS-CoV-2 genomes of the Delta lineage sequenced from COVID-19-positive samples collected during the same timeframe as the pet samples (Figure 1). Additionally, pets from the same household were found to be infected with the same SARS-CoV-2 lineages (two co-habiting pairs of dogs, infected with AY.25.1 and AY.117, respectively; and one dog and cat pair infected with B.1.575 [20]), indicating likely exposure to the same human source or viral sharing between pets. Furthermore, due to robust genomic sequencing efforts in Arizona to track variants associated with human cases [20,21], the lineages identified from viral sequencing of the pet samples (B.1.575, B.1.617.2, and AY.103) also matched the lineages identified in the SARS-CoV-2-positive samples from their pet owners. Figure 1 displays a maximum likelihood phylogeny of Delta-variant SARS-CoV-2 genomes from pets enrolled in AZCPP, pet owners [with available viral sequence data], and 35 other SARS-CoV-2 genomes derived from human COVID-19-positive samples collected within the same geographic region and timeframe of the study. The phylogeny highlights the genomic relationship between the two dogs from household 1 (HH1) infected with AY.25 and the dog and pet owner from household 2 (HH2) infected with AY.103. Only one mutation separates the genomes from the two dogs from HH1 and there are zero mutations between the genomes from the dog and pet owner from HH2.

### 3.3. Risk Factor Analysis

Immunocompromised pets were 1.83 times more likely to test positive for SARS-CoV-2 (*p* = 0.03) than non-immunocompromised pets (Table 2). Additionally, pets living in households where more than one individual in the house tested positive for COVID-19 were significantly more likely to test positive for SARS-CoV-2 (32/61; 52.5%; *p* = 0.0001) than pets living in households where only one individual tested positive for COVID-19. Pets sampled within 14 days of the human case’s symptom onset were also significantly more likely to test positive for SARS-CoV-2 (8/26; 30.8%; *p* = 0.01). There were no statistically significant associations among the remaining factors investigated. The days between case onset and sample collection were determined by the average from a literature review [26,27].

## 4. Discussion

We found that a relatively high proportion (36.4%) of the pets enrolled in AZCPP had evidence of infection with SARS-CoV-2. These findings reveal a high likelihood of viral transmission in households when pets lived in homes in which multiple humans had tested positive for SARS-CoV-2. Pets were more likely to test RT-PCR-positive for SARS-CoV-2 if the samples were collected 14 or fewer days after the person with COVID-19′s symptom onset, consistent with the known viral shedding period of less than 5 days [28]. More than one person testing positive in the household was shown to increase the risk of a pet testing positive, potentially via a higher number of virus particles in the air/proximal to the pet, and/or prolonged exposure to the virus [29,30]. Our findings on the risk factors for SARS-CoV-2-positive pets is mostly consistent with previous studies that found no association between the SARS-CoV-2 status of the animal and age, sex, or health status; however, the pets in our study appeared to be at higher risk for infection with SARS-CoV-2 if they were immunocompromised [31]. Pet owners who have immunocompromised pets should take precautions to reduce the risk to their pets.

Specific interactions such as petting/cuddling between pets and ill owners did not appear to increase the risk; however, cohabitation between people and pets is inherently a risk, since cats and dogs are susceptible to airborne transmission [32]. In scenarios where there is only one caretaker of pets or complete separation between ill persons and pets is not feasible, reducing contact in other practical ways can be achieved to potentially reduce the likelihood of spreading SARS-CoV-2 to pets in the household. For example, although our study did not show a statistically significant difference between sleeping in the same bed or not, other studies have identified this as a risk factor and could be taken into consideration when reducing contact with pets [32,33,34]. Additionally, food sharing was found to be a risk factor for infection among pets sampled in Latin America [35].

The majority of the sampled animals were asymptomatic, consistent with other studies [27,36], and this emphasizes the importance of active surveillance studies as well as the education of pet owners and veterinarians regarding the possibility of infection in animals. Symptom variability between dogs and cats in our study was similar to other studies in that infections typically appear asymptomatic in dogs, while clinical signs of respiratory disease tend to be mild or moderate in cats [37]. Consistent with other studies, the majority of our SARS-CoV-2-positive pets (70%) were asymptomatic [26,31]. All ill pets in our study survived/recovered from their mild illness, although severe disease and death due to SARS-CoV-2 infection in animals has been reported [3,38]. Interestingly, the majority of the seropositive pets in our study were dogs (22/31), yet cats appeared to present higher seroprevalence with higher titers of neutralizing antibodies; this finding may represent the timing of sample collection or the particular population of pets we sampled and would need other studies to come to a more definite conclusion [39].

Although not statistically significant in our study, the number of pets in a household may still play a role in the risk for SARS-CoV-2 infection in pets; the likelihood of infection from one cat to another cat has been considered to be the same as that of one person to another person, and inter-species transmission is roughly half the probability [40]. Cat-to-cat transmission of SARS-CoV-2 has been documented [41], but we could not prove these more nuanced transmission events in our study. Our study did find a higher rate of positivity for other pets in multi-pet households (10/12) than another study that tested multi-pet households (0/3) [27]. This difference could be due to changes in the SARS-CoV-2 virus itself, interactions the pets had with positive household members, interactions the pets had with a positive pet, and/or other factors. Variation in the types of interactions between pets in a household could play a role in pet-to-pet transmission. While we cannot rule out the likelihood that the pets in our study all became infected through interaction with people, further work is needed to identify pet-to-pet transmission.

Our study did, however, show potential transmission between dogs living in the same household, as well as from pet owner to dog. In addition to the sequencing conducted on the SARS-CoV-2-PCR-positive pet samples, including dogs cohabitating the same household, we were able to obtain viral genomes from pet owners from two households. The phylogenetic analysis of the SARS-CoV-2 Delta variant infecting pets reported in this study (Figure 1) revealed evidence of likely dog-to-dog transmission (HH1) and pet owner-to-dog transmission (HH2); 0–1 mutations separated viral genomes in these instances. We previously described a cluster involving a dog, cat, and pet owner from the same household in which identical viral genomes of the B.1.575 lineage were identified [21], suggesting cross-species transmission with suspected directionality from the owner to at least one pet. The additional findings from the larger AZCPP study add to the knowledge base that SARS-CoV-2 can be transmitted from infected people to pets and from animal to animal.

One limitation to evaluating the effect of household risk based on vaccination status in our study was the inability to fully evaluate human immunity; we did not obtain dates or proof of vaccination. This is important because studies show that fully vaccinated patients have shorter viable viral shedding periods compared to unvaccinated or partially vaccinated patients [42,43]. We could assume, then, that fully vaccinated members of the household could pose less of a risk of infecting their pets. In addition, although type of housing was used as a proxy for the amount of space and potential exposure to SARS-CoV-2 virus in the air, the exact square footage for each residence sampled was not obtained. Indoor air space is one factor that has been studied to determine risk for the infection and spread of SARS-CoV-2; these more nuanced environmental factors were not practical for us to fully explore in this study [29,30]. Another limitation, with regard to laboratory testing, is that it was not possible to obtain every sample type from every pet due to the pet experiencing stress or discomfort. It is possible that some pets tested negative but may have actually been positive had we been able to collect each sample type from them (nasal vs. rectal swab, or blood sample vs. swabs); for example, for pets sampled later in their exposure period, we would have a higher chance of the pet testing seropositive than PCR-positive due to how long the virus was shed and, thus, a blood sample would be best to collect [28,31,39]. Some pets were sampled outside of a more ideal 14-day sampling window due to schedule coordination between the pet owners and sampling staff. Public health departments, researchers, animal agriculture agencies, and/or private practice veterinarians can maximize timing by planning ahead and increasing the convenience of scheduling (such as having study staff visit a person’s home, as in our study). Serial serology, although of benefit in other studies [26], was not practical in our study based on staff availability. Serial testing of dogs and cats in an Italian study found long-term persistence of neutralizing antibodies: up to 10 months in some of the animals; however, the window for sampling should be much sooner in order to maximize the likelihood of detecting circulating antibodies that may exist following an infection [41].

A collaborative One Health approach is critical during animal SARS-CoV-2 investigations to guide recommendations on how positive humans should interact with animals. When time is limited, groups of interest such as persons with variants of concern or pets with severe illness can be sampled in close geographic areas. If resources are limited, health departments or veterinarians should prioritize swabs for PCR testing if performed early in the identification of the positive human case; if sampling cannot occur in a timely manner, serum should be collected for the analysis of circulating antibodies, which can be detected several weeks post-infection. Outside of enhanced surveillance programs, SARS-CoV-2 sampling may need to occur on a case-by-case basis if prioritization is needed, such as for severe cases, newly circulating variants in the human population, or newly identified susceptible species. Once these One Health surveillance systems are put into place, they should be sustained and maintained with protocols, funding, and frequent communication between collaborating sectors such as animal and human health agencies.

Further surveillance studies, including genomic analyses, are needed to characterize how new SARS-CoV-2 variants impact animals and to understand risks for infection and spillover in susceptible species.

## Figures and Tables

**Table 1:** Characteristics of dogs and cats sampled in Arizona (N = 110).

Characteristic	Number of Pets (%)
Species	
Dog	71 (64.5%)
Cat	39 (35.5%)
Breed/Breed Group	
Dog (n = 71)	
Terrier	14 (19.7%)
Herding	11 (15.5%)
Toy	11 (15.5%)
Sporting	8 (11.3%)
Mixed breed	8 (11.3%)
Working	7 (9.9%)
Hound	5 (7.0%)
Non-sporting	4 (5.6%)
Foundation stock service	3 (4.2%)
Cat (n = 39)	
Domestic Shorthair	28 (71.8%)
Domestic Longhair	5 (12.8%)
Siamese	3 (7.7%)
American Curl	1 (2.6%)
Devon Rex	1 (2.6%)
Maine Coon	1 (2.6%)
Age	3 months–15 years (mean 5.8 years)
Range	
<1 year (pediatric)	15 (13.6%)
1–8 years (adult)	63 (57.3%)
>8 years (senior)	32 (29.1%)
Sex	
Male	60 (54.5%)
Female	50 (45.5%)
Spay/Neuter Status	
Spayed/neutered	94 (85.5%)
Not spayed/neutered	16 (14.5%)
SARS-CoV-2 laboratory testing	
Negative	70 (63.6%)
Positive	40 (36.4%)
PCR+ only	9
Seropositive only	21
PCR+ and seropositive	10
Health Status	
Asymptomatic	77 (70%)
Recent clinical signs	17 (15.5%)
Current clinical signs	16 (14.5%)
Clinical signs ^1^ (n = 33)	
Sneezing	26 (78.8%)
Lethargy	15 (45.5%)
Ocular discharge	14 (42.4%)
Nasal discharge	13 (39.4%)
Coughing	13 (39.4%)
Difficulty breathing	8 (24.2%)
Anorexia/inappetence	6 (18.2%)
Fever	4 (12.1%)
Vomiting	2 (6.1%)
Diarrhea	2 (6.1%)
Time (in days) between case onset and pet sample collection	4–114 (mean 31, median 17)

^1^ More than one clinical sign may have been reported for some pets.

**Table 2:** Associated factors for SARS-CoV-2-positive dogs and cats in Arizona. The * denotes statistical significance.

Variable	Number of Positive Pets/ Total (%)	Relative Risk (95% Confidence Interval); *p*-Value (Fisher Exact)
Cat	13/39 (33.3%)	RR 0.88 (0.51–1.50)
Dog	27/71 (38.0%)	
Pediatric/old (<1 or >8 yrs.)	14/48 (29.2%)	RR 1.22 (0.92–1.61)
Adult (1–8 years old)	26/62 (41.9%)	
Male	17/50 (34.0%)	RR 0.89 (0.54–1.47)
Female	23/60 (38.3%)	
Spayed/neutered	31/94 (33.0%)	RR 0.58 (0.35–0.99); *p* = 0.09
Not spayed/neutered	9/16 (56.3%)	
Pet immunocompromised	14/25 (56.0%)	RR 1.83 (1.14–2.94); *p* = 0.03 *
Pet not immunocompromised	26/85 (30.6%)	
Pet ever symptomatic	13/33 (39.4%)	RR 1.12 (0.67–1.89)
Pet asymptomatic	27/77 (35.1%)	
More than one individual in household tested positive	32/61 (52.5%)	RR 3.21 (1.63–6.33); *p* = 0.0001 *
Only one individual in household tested positive	8/49 (16.3%)	
Multiple pets residing in the household	34/95 (35.8%)	RR 0.89 (0.46–1.76)
Single pet household	6/15 (40.0%)	
Household residence type:		RR 0.97 (0.38–2.45)
Private home (free-standing)/townhouse	37/102 (36.3%)	
Apartment/condo	3/8 (37.5%)	
Household risk (vaccination status):		RR 0.77 (0.47–1.27)
Low/Medium	20/62 (32.3%)	
High	20/48 (41.7%)	
PCR samples:		RR 3.54 (1.36–9.22); *p* = 0.01 *
≤14 days between case onset and pet sample collection	8/26 (30.8%)	
>14 days between case onset and pet sample collection	6/69 (8.7%)	
Virus neutralization (serology) samples:		RR 3.30 (0.86–12.64)
≤30 days between case onset and pet sample collection	19/46 (41.3%)	
>30 days between case onset and pet sample collection	2/16 (14.3%)	
Pet contact w/symptomatic case:		RR 1.78 (0.98–3.26)
Slept in same bed	30/69 (43.5%)	
Did not sleep in same bed	10/41 (24.4%)	
Licking face and/or hands	17/49 (34.7%)	RR 0.92 (0.56–1.52)
Did not lick face and/or hands	23/61 (37.7%)	
Sharing food and/or utensils	0/5	RR 0.37 (0.06–2.33)
Not sharing food and/or utensils	40/105 (38.1%)	
Providing medical care to pet	4/7 (57.1%)	RR 1.63 (0.82–3.27)
Not providing medical care to pet	36/103 (35.0%)	
Taking walks (dogs only)	13/33 (39.4%)	RR 1.07 (0.59–1.94)
Not taking walks (dogs only)	14/38 (36.8%)	
Lying on couch together/pet sits on lap	27/84 (32.1%)	RR 0.64 (0.39–1.05)
No lying on couch together/pet does not sit on lap	13/26 (50.0%)	
Cleaning pet waste	11/26 (42.3%)	RR 1.22 (0.72–2.10)
No cleaning pet waste	29/84 (34.5%)	
Petting/cuddling	35/100 (35.0%)	RR 0.70 (0.36–1.37)
No petting/cuddling	5/10 (50.0%)	

## Data Availability

The data presented in this study are available on request from the corresponding author. Genomes have been published to GISAID. Accession IDs are included in Supplementary Table S1.

## Supplementary Materials

The following supporting information can be downloaded at: https://www.mdpi.com/article/10.3390/pathogens13060466/s1. Table S1: Metadata and GISAID Accession ID numbers for SARS-CoV-2 genomes obtained from companion animal samples sequenced in this study, available genomes from pet owners and 35 human background samples (obtained through GISAID) utilized in the maximum likelihood phylogenetic tree (Figure 1) built with IQ-Tree and visualized using MicrobeTrace.
