# Supplementary material for: Investigation of SARS-CoV-2 Infection among Companion Animals in Households with Confirmed Human COVID-19 Cases
Source: Pathogens. 2024 Jun 1;13(6):466. doi: 10.3390/pathogens13060466 (PMC11206992; doi:10.3390/pathogens13060466)
Supplement: Supplementary file 1 [file pathogens-13-00466-s001.zip › pathogens-3001942-supplementary.pdf]

Supplemental Table S1: Metadata and GISAID Accession ID numbers for SARS-CoV-2 genomes obtained from companion animal samples sequenced in this study, available genomes from pet owners and 35 human background samples (obtained through GISAID) utilized in the maximum likelihood phylogenetic tree+ (Figure 1) built with IQ-Tree and visualized using MicrobeTrace.

Sample ID	GISAID ID	Collection Date	County	Pangolin Lineage
HH1_Canine_1	EPI_ISL_12543433	11/17/2021	Maricopa	AY.25.1
HH1_Canine_2	EPI_ISL_12543434	11/17/2021	Maricopa	AY.25.1
HH2_Canine	EPI_ISL_12543430	11/8/2021	Maricopa	AY.103
HH2_Human	EPI_ISL_6584142	11/4/2021	Maricopa	AY.103
HH3_Canine	EPI_ISL_12543431	12/3/2021	Coconino	AY.103
HH4_Canine	EPI_ISL_12543432	11/17/2021	Maricopa	B.1.617.2
HH5_Feline	Not shown	12/2/2021	Navajo	B.1.617.2
HH6_Canine	EPI_ISL_12543435	12/10/2021	Maricopa	AY.117
HH6_Canine	Not shown	12/10/2021	Maricopa	Unassigned, mutations present indicate AY.117
AZ_1	EPI_ISL_7898362	12/13/21	Coconino	B.1.617.2
AZ_2	EPI_ISL_9397021	12/22/21	Maricopa	AY.25.1
AZ_3	EPI_ISL_7735329	12/8/21	Coconino	AY.103
AZ_4	EPI_ISL_7269972	11/23/21	Maricopa	AY.25.1
AZ_5	EPI_ISL_7745577	10/22/21	Maricopa	AY.103
AZ_6	EPI_ISL_6863114	11/6/21	Maricopa	B.1.617.2
AZ_7	EPI_ISL_6573145	11/6/21	Maricopa	AY.117
AZ_8	EPI_ISL_9396757	12/13/21	Maricopa	AY.103
AZ_9	EPI_ISL_6573131	11/9/21	Maricopa	AY.103
AZ_10	EPI_ISL_11100750	12/7/21	Maricopa	AY.103
AZ_11	EPI_ISL_7735466	12/8/21	Maricopa	AY.25.1
AZ_12	EPI_ISL_7659415	10/15/21	Maricopa	AY.103
AZ_13	EPI_ISL_6228296	11/2/21	Maricopa	AY.103
AZ_14	EPI_ISL_6074997	10/29/21	Maricopa	AY.103
AZ_15	EPI_ISL_8475090	12/7/21	Coconino	AY.103
AZ_16	EPI_ISL_8373877	12/23/21	Maricopa	AY.103
AZ_17	EPI_ISL_8020629	12/16/21	Maricopa	AY.103
AZ_18	EPI_ISL_13197479	11/18/21	Maricopa	AY.25.1
AZ_19	EPI_ISL_13197548	12/13/21	Maricopa	AY.103
AZ_20	EPI_ISL_7495781	12/3/21	Maricopa	AY.103
AZ_21	EPI_ISL_7983581	12/16/21	Maricopa	AY.103
AZ_22	EPI_ISL_13041861	11/15/21	Maricopa	AY.103
AZ_23	EPI_ISL_7674907	12/7/21	Maricopa	AY.103
AZ_24	EPI_ISL_8373883	12/23/21	Maricopa	AY.103
AZ_25	EPI_ISL_7845109	12/12/21	Maricopa	AY.25.1
AZ_26	EPI_ISL_7735448	11/28/21	Maricopa	AY.103
AZ_27	EPI_ISL_11569217	12/29/21	Maricopa	AY.103

AZ_28	EPI_ISL_7462551	12/1/21	Coconino	AY.103
AZ_29	EPI_ISL_6666926	11/9/21	Maricopa	AY.103
AZ_30	EPI_ISL_8163222	12/21/21	Maricopa	AY.103
AZ_31	EPI_ISL_5644349	10/15/21	Coconino	AY.103
AZ_32	EPI_ISL_5644433	10/19/21	Maricopa	AY.103
AZ_33	EPI_ISL_6572738	11/7/21	Maricopa	AY.103
AZ_34	EPI_ISL_5644501	10/19/21	Maricopa	AY.25.1
AZ_35	EPI_ISL_7745619	10/25/21	Maricopa	AY.103
